# Supplementary material for: Hydrotalcite-Embedded Magnetite Nanoparticles for Hyperthermia-Triggered Chemotherapy
Source: Nanomaterials (Basel). 2021 Jul 9;11(7):1796. doi: 10.3390/nano11071796 (PMC8308439; doi:10.3390/nano11071796)
Supplement: Supplementary file 1 [file nanomaterials-11-01796-s001.zip › nanomaterials-1271827-supplementary.pdf]

nanomaterials

Supplementary Materials

Hydrotalcite-Embedded Magnetite Nanoparticles for Hyperthermia-Triggered Chemotherapy

Konstantinos Simeonidis [1,2,*], Efthimia Kaprara [1], Pilar Rivera-Gil [3], Ruixue Xu [3], Francisco J. Teran [4,5], Evgenios Kokkinos [2], Athanassios Mitropoulos [6], Nikolaos Maniotis [7] and Lluis Balcells [8]

1 Department of Chemical Engineering, Aristotle University of Thessaloniki, 54124 Thessaloniki, Greece; ksime@physics.auth.gr; kaprara@auth.gr
2 Ecoresources P.C., Giannitson-Santaroza Str. 15-17, 54627 Thessaloniki, Greece; kokkinos@ecoresources.gr
3 Integrative Biomedical Materials and Nanomedicine Lab, Universitat Pompeu Fabra, 08003 Barcelona, Spain; pilar.rivera@upf.edu (P.R.-G.); ruixue.xu01@estudiant.upf.edu (R.X.)
4 IMDEA-Nanociencia, Ciudad Universitaria de Cantoblanco, 28049 Madrid, Spain; francisco.teran@imdea.org
5 Nanobiotecnología (iMdea-Nanociencia), Unidad Asociada al Centro Nacional de Biotecnología (CSIC), 28049 Madrid, Spain
6 Hephaestus Advanced Laboratory, Department of Chemistry, International Hellenic University, 65404 Kavala, Greece; amitrop@chem.ihu.gr
7 Department of Physics, Aristotle University of Thessaloniki, 54124 Thessaloniki, Greece; nimaniot@physics.auth.gr
8 Institut de Ciencia de Materials de Barcelona, CSIC, 08193 Bellaterra, Spain; balcells@icmab.es
* Correspondence: ksime@physics.auth.gr

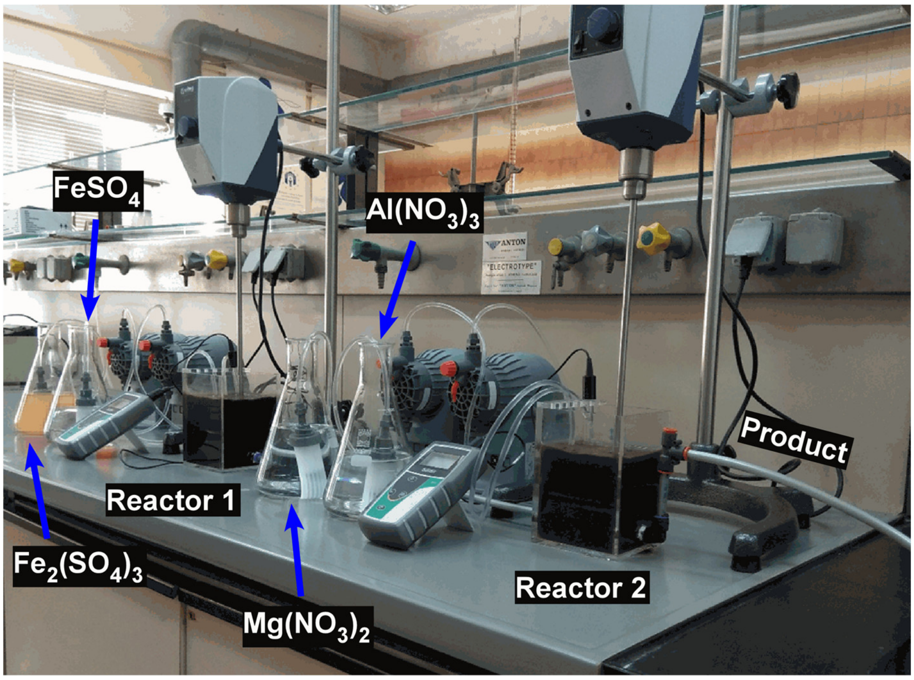

Figure S1. Laboratory setup for the continuous-flow production of the hydrotalcite/magnetite nanocomposites.

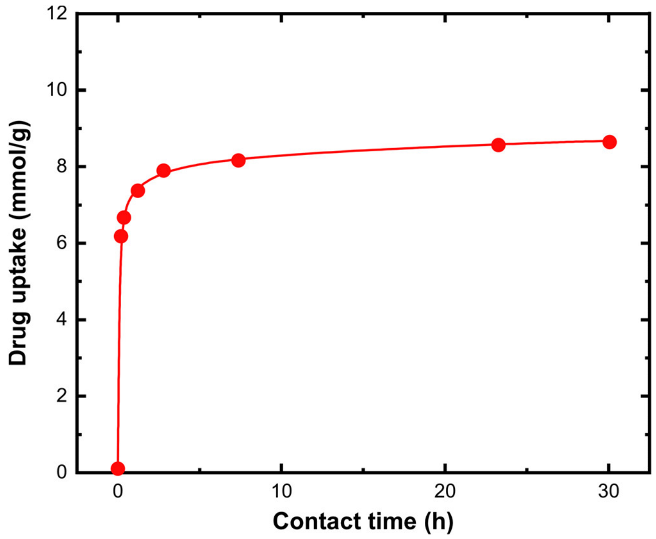

Figure S2. Kinetic of drug loading in the structure of the MGT-35. Experiment was performed in a phosphate-buffered saline (PBS) solution adjusted to pH 9 with 0.5 mM of 5-fluorouracil and 2 g/L of dispersed nanomaterial.

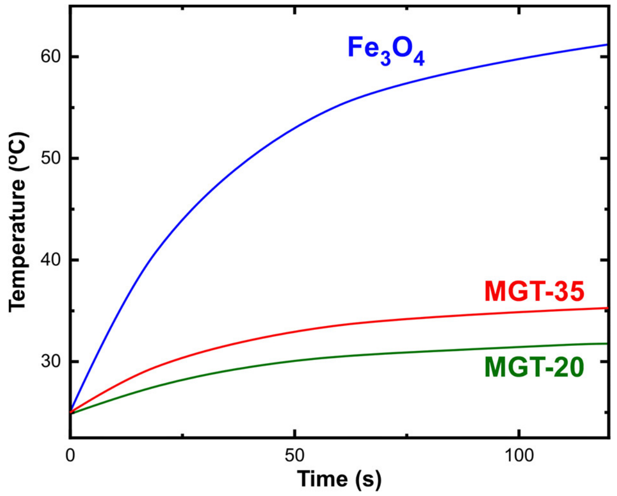

Figure S3. Temperature increase during AC field application (strength 24 kA/m, frequency 765 kHz) on an aqueous dispersion (2 g/L) of MGT-20 and MGT-35, and a corresponding Fe_3O_4 nanoparticles reference sample.

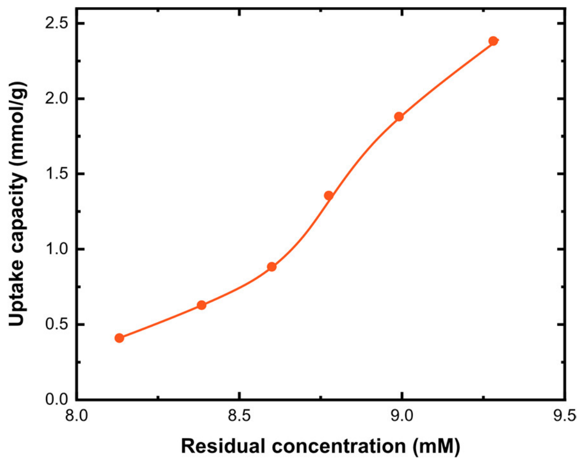

Figure S4. Uptake capacity versus residual 5-fluorouracil concentration for Magnother-35. Experiments were carried out in a phosphate-buffered saline (PBS) solution adjusted to pH 9.

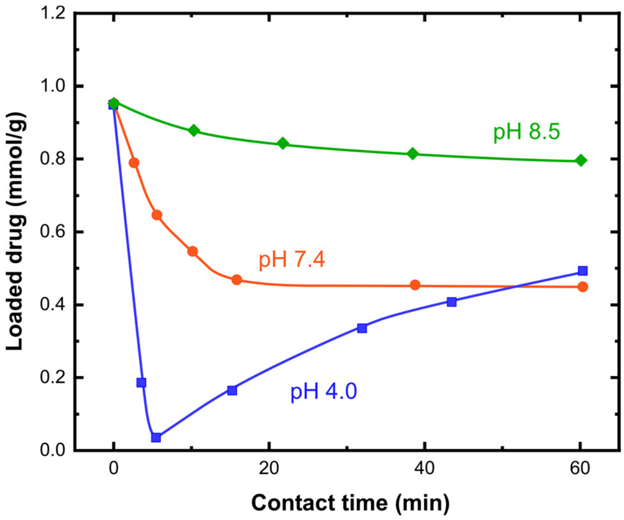

Figure S5. Time dependent leaching behavior of 5-fluorouracil from initially loaded MGT-35 (~1 mmol/g) dispersed in a phosphate-buffered saline (PBS) solution adjusted to various pH values.

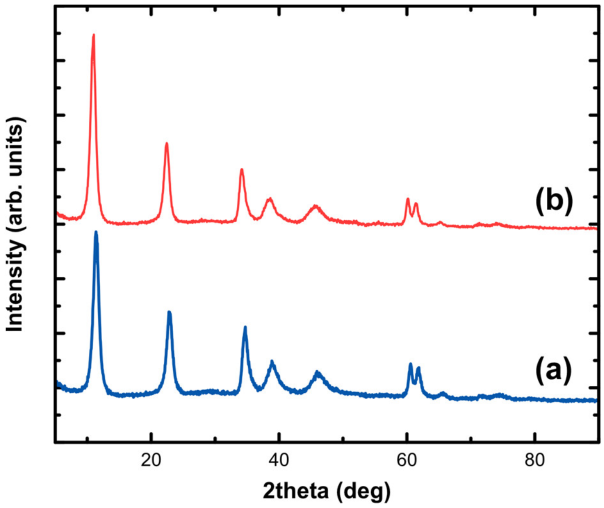

Figure S6. XRD diagrams of pure hydrotalcite before (**a**) and after loading with 5-fluorouracil (**b**).

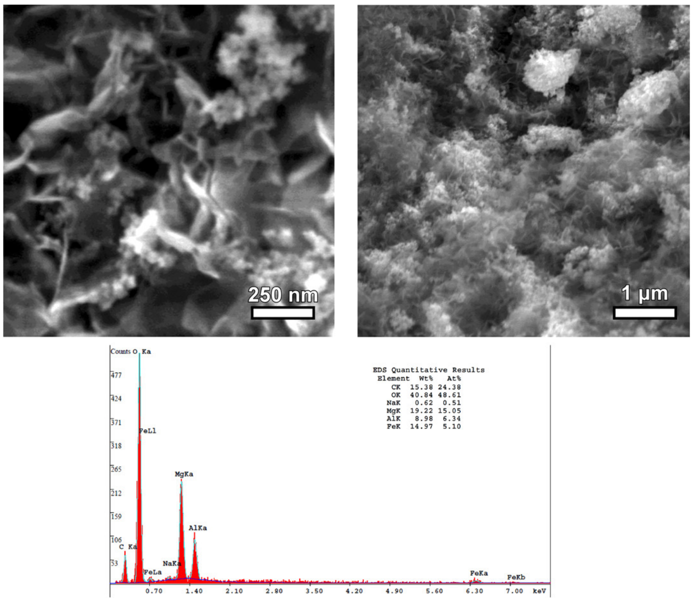

Figure S7. Scanning electron microscopy images and elemental analysis of sample MGT-35.
